# *Bacteroides cellulosilyticus*-derived 2-hydroxyphenylacetic acid rectifies hepatic lipid homeostasis in MASLD by targeting the PPARγ-CD36 axis

**DOI:** 10.1080/19490976.2026.2725392

**Published:** 2026-09-01

**Authors:** Kaiwei Chen, Zizhen Yang, Jixing Peng, Chunyan Liu, Ying Yu, Xinchi Cai, Bei Liu, Shuao Li, Tiantian Chen, Samil Jung, Yu Tian, Qianyue Xu, Xing Rao, Zhinan Wu, Haoyu Wang, Yan Di, Linna Wang, Jianxun Wang, Myeong-sok Lee, Yuanqiang Zou, Ningning He, Shangyong Li

**Affiliations:** a School of Basic Medicine, Qingdao Medical College, Qingdao University, Qingdao, China; b Yellow Sea Fisheries Research Institute, Chinese Academy of Fishery Sciences, Qingdao, China; c Molecular Cancer Biology Laboratory, Cellular Heterogeneity Research Center, Department of Biosystem, Sookmyung Women's University, Seoul, Korea; d State Key Laboratory of Genome and Multi-omics Technologies, BGI Research, Shenzhen, China; e College of Life Sciences, Northwest University, Xi'an, China; f College of Life Science, University of Chinese Academy of Sciences, Beijing, China; g Shenzhen Key Laboratory of Human Commensal Microorganisms and Health Research, BGI Research, Shenzhen, China; h BGI Precision Nutrition, Shenzhen, China

**Keywords:** MASLD, *Bacteroides cellulosilyticus*, 2-Hydroxyphenylacetic acid, CD36 palmitoylation, lipid homeostasis

## Abstract

The gut microbiota plays an important role in the occurrence and development of metabolic dysfunction-associated steatotic liver disease (MASLD), but the specific molecular mechanisms involved have not been fully elucidated. In this study, human cohort studies were performed to identify that the relative abundance of *Bacteroides cellulosilyticus* (*B. cellulosilyticus*) was significantly decreased in patients with MASLD. Through the integration of metagenomic and metabolomic analyses, it was confirmed that *B. cellulosilyticus* and its metabolite 2-hydroxyphenylacetic acid (2HPAA) are key factors regulating the occurrence and development of MASLD. Single-cell sequencing and lipidomic analyses revealed that 2HPAA can enter the liver through the enterohepatic circulation to exert regulatory effects. Specifically, 2HPAA inhibits the peroxisome proliferator-activated receptor *γ* (PPARγ) signaling pathway, thereby suppressing the expression of the fatty acid transporter CD36. Meanwhile, 2HPAA regulates lipid metabolism in hepatocytes by significantly enhancing palmitate conversion efficiency and inhibiting CD36 palmitoylation. This dual regulatory effect on CD36 expression and palmitoylation can reduce lipid accumulation in hepatocytes and ultimately alleviate MASLD progression. These findings reveal the mechanism by which *B. cellulosilyticus* and 2HPAA alleviate MASLD by targeting the PPARγ-CD36 pathway. This work provides a new perspective for the study of gut microbiota-host interactions in regulating liver diseases.

## Graphical abstract

**Figure:**
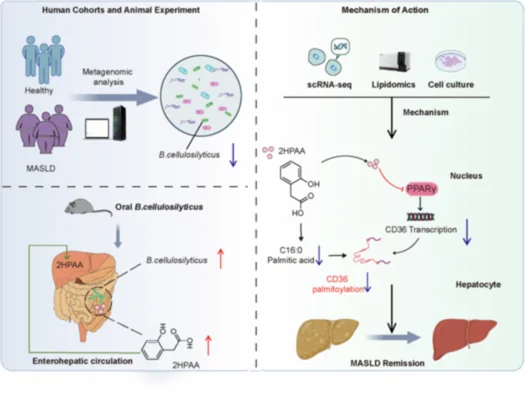

## Introduction

Metabolic dysfunction-associated steatotic liver disease (MASLD) has emerged as the most widespread chronic liver disease globally, impacting up to 38% of adults worldwide.^1^ It encompasses a wide spectrum of liver injuries, ranging from hepatic steatosis, metabolic dysfunction-associated steatohepatitis (MASH), fibrosis, and cirrhosisto MASLD-associated hepatocellular carcinoma (MASLD-HCC).^2^ ^,^ ^3^ Additionally, MASLD often co-occurs with other metabolic disorders, such as insulin resistance (IR), type 2 diabetes mellitus (T2DM), and cardiovascular disease (CVD), thereby increasing the risk of adverse clinical outcomes.^4^ Nevertheless, the complex and diverse etiological and pathological factors underlying the development and progression of MASLD have yet to be fully elucidated.^5^ Moreover, to date, no anti-MASLD drugs have obtained international approval.^6^

The gut microbiota and its metabolites play a crucial role in MASLD pathogenesis.^7^ ^,^ ^8^ It is well established that specific pathogenic bacteria are deeply involved in MASLD progression by actively driving hepatic *de novo* lipogenesis, promoting inflammation, and accelerating the formation of fatty liver.^9^ Nevertheless, although gut microbiota dysbiosis is a common characteristic of MASLD patients, the associated functional mechanisms and host responses of specific microbial species and metabolites remain insufficiently characterized.^10^ Within the gut microbiota ecosystem, the genus *Bacteroides* represents a predominant portion of the commensal microbiota and plays a crucial role in the fermentation of complex dietary carbohydrates.^11-13^ Specifically, *Bacteroides cellulosilyticus* (*B.cellulosilyticus*) is distinguished by its exceptional metabolic specialization.^14^ While various beneficial microbes decrease during MASLD progression, our preliminary large-scale cohort screening identified *B. cellulosilyticus* as a topologically central keystone species whose depletion is strongly linked to metabolic disorders. This species encodes an extensive repertoire of carbohydrate-active enzymes (CAZymes) and polysaccharide utilization loci, surpassing most other known gut symbionts in its capacity to hydrolyze diverse complex polysaccharides, including cellulose, xylan, and pectin.^15^ ^,^ ^16^ Despite these extensive enzymatic capabilities, the potential probiotic role of *B.cellulosilyticus* in MASLD and the underlying mechanisms influencing liver homeostasis remain to be fully elucidated.

The gut-liver axis serves as a critical bidirectional conduit connecting the intestine and liver, wherein gut microbiota-derived metabolites function as pivotal signaling molecules regulating hepatic homeostasis and inflammatory responses.^17^ ^,^ ^18^ Aromatic amino acid derivatives serve as pivotal signaling molecules that cross the intestinal barrier to act on host nuclear receptors or metabolic sensors, thereby regulating immune and metabolic homeostasis in distal organs.^19-21^ Previous studies have indicated that tyrosine metabolites, such as 4-hydroxyphenylacetic acid (4HPAA) and 3-hydroxyphenylacetic acid (3HPAA), reverse hepatic steatosis by activating the SIRT1 signaling pathway or downregulating hepatic histone acetylation levels.^22^ ^,^ ^23^ However, the role and mechanism of phenylalanine derivative, 2-hydroxyphenylacetic acid (2HPAA), in anti-MASLD remain unclear.

In this study, a series of *in vivo* and *in vitro* analyses identified that *B. cellulosilyticus* and its derived 2HPAA exerts a significant preventive effect on MASLD. Meanwhile, we also investigated the possible mechanism by which 2HPAA exerts its lipid homeostasis effect *via* the PPARγ-CD36 axis.

## Materials and methods

### Materials

The normal chow diet (NCD, #1025) and a high-fat diet (HFD, #H10060) were purchased from Beijing Huafukang Biotechnology Co., Ltd. (Beijing, China), and the detailed composition of the diets is presented in Table S1. The 2HPAA (H49804) with a purity  ≥ 98% was purchased from Sigma–Aldrich (St. Louis, MO, USA). Rosiglitazone (R128083) and methoxy-polyethylene glycol-maleimide (mPEG-mal, MW 10000, M796652) were purchased from Aladdin (Shanghai, China). *B. cellulosilyticus* AF17-25 was isolated and cultured by BGI-Shenzhen (Shenzhen, China). Standard samples of L-phenylalanine (DRE-C16055100) and L-tyrosine (DRE-C17896005) were obtained from Dr. Ehrenstorfer (Augsburg, Germany). Standard samples of 2HPAA (H49804) were purchased from Sigma–Aldrich (St. Louis, MO, USA). Additional materials are listed in the Supplemental Materials and Methods.

### Animal study

Animal procedures were approved by the Ethics Committee of Medical College of Qingdao University (QDU-AEC-2025588) following the guidelines of the National Institutes of Health (NIH) and the ARRIVE guidelines 2.0. Six-week-old C57BL/6J mice (male, 18‒20 g) were maintained in specific pathogen-free (SPF) conditions with controlled temperature (24 ± 2 °C), relative humidity (50 ± 5%), and a 12-h light/dark cycle. After 1 week of acclimatization, the mice were randomly allocated to experimental groups (*n* = 8). The mice were fed either an HFD or an NCD. For intragastric administration, 100 μL of phosphate-buffered saline (PBS), *B. cellulosilyticus* (1 × 10^10^ CFU/mL), Rosiglitazone (1  mg/kg), or 2HPAA (150  mg/kg) by oral gavage for the indicated duration. At the end of the experiment, following a 12-hour fasting period, the mice were deeply anesthetized with inhalational isoflurane (R510-22-10, RWD Life Science, Shenzhen, China) using an R500 small animal anesthesia machine (RWD Life Science, Shenzhen, China). Blood samples were collected via orbital sinus bleeding from the mice under deep anesthesia. Immediately following blood collection, the mice were euthanized by cervical dislocation while still under deep anesthesia. All anesthesia and euthanasia procedures were conducted in strict accordance with the American Veterinary Medical Association (AVMA) Guidelines for the Euthanasia of Animals. Refer to Supplemental Materials and Methods for relevant organization collection and daily data recording.

### Population-based cohort

The metagenomic profiles and clinical metadata used in this study were obtained from the curatedMetagenomicData R package. Samples with BMI records were selected, while those in disease states, with low sequencing quality, or missing key clinical information were excluded. Simultaneously, items with a low proportion of samples were removed to minimize batch effects. After these stringent filters, 2,800 high-quality samples from healthy individuals were included in subsequent analyses. Samples with no assigned reads for *B. cellulosilyticus* represented observational zeros, and their untransformed relative abundance was set to 0 after total-sum normalization. When a logarithmic transformation was needed for statistical testing, a pseudocount of 10^−6^ was incorporated before the log_10_ transformation. Detection prevalence was defined as the percentage of samples whose untransformed relative abundance was greater than zero. All prevalence and baseline abundance results are reported using untransformed data. The raw sequencing data accession numbers for all disease cohorts are provided in Table S2.

### Bacterial culture and genomic analysis

*B. cellulosilyticus* (accession number AF17-25) was isolated from a fecal sample of a healthy female donor through the BGI human gut bacterial culture program. Fresh fecal samples were processed under anaerobic conditions, serially diluted, and cultured using media optimized for human gut bacteria, with single colonies purified by repeated streaking. The purified isolate was identified and verified as *B. cellulosilyticus* based on 16S rRNA gene sequencing and whole-genome-based taxonomic analysis. Strain AF17-25 has been deposited in BGI Research under the Cultivated Genome Reference (CGR) project.^24^ *B. cellulosilyticus*, verified by 16S rRNA sequencing, was cultured in MPYG broth.^25^ Fresh suspensions (10^10^ CFU/mL in sterile reduced PBS buffer) were prepared by centrifugation (3,000  rpm, 4 °C, 5 min). Gene prediction and preliminary annotation of the *B. cellulosilyticus* AF17-25 genome were performed using Prokka. The genome assembly and annotation data of AF17-25 are publicly available through CNGBdb/CNSA under Project ID CNP0000126. Refer to Supplemental Materials and Methods for further analysis.

### Construction of *aroB*-deficient *B. cellulosilyticus* strain

To target the *aroB* gene, 500 bp upstream and downstream homology arms were amplified and cloned into the pGERM suicide vector. The recombinant plasmid was then transformed into *E. coli* donor strain. Bacterial conjugation was performed by mixing the *E. coli* donor (cultured in LB medium supplemented with ampicillin) and *B. cellulosilyticus* recipient (cultured in MYPG medium) at a 1:1 ratio. The mixed bacterial suspension was incubated at 37 °C for 8 h, after which 200  μL of the mixture was spread onto erythromycin-selective agar plates. After 48 h of anaerobic incubation, erythromycin-resistant colonies were selected to extract PCR and agarose gel electrophoresis to verify the disruption of *aroB* gene. Refer to Supplemental Materials and Methods for specific steps,

### Metagenomic sequencing

Genomic DNA was extracted from fecal samples using the MagPure Stool DNA KF Kit B (Magen Biotechnology). Shotgun metagenomic sequencing was performed on the BGISEQ-500 platform (BGI) to generate 100 bp paired-end reads. The raw reads were quality-filtered and trimmed using fastp, retaining high-quality reads (Phred score ≥ 20, length ≥ 51 bp). Host reads were removed by mapping to the mouse reference genome (GRCm38) using Bowtie2. Taxonomic classification was performed using Kraken2 and Bracken. A non-redundant gene catalog was constructed and quantified via MetaWibele, with functional annotation using eggNOG-mapper.

### Bulk RNA sequencing

Total RNA was extracted from liver tissue using the Sparkjade Total RNA Extraction Kit and assessed via an Agilent 2100 Bioanalyzer. mRNA was enriched using Oligo (dT) beads to construct cDNA libraries, which were sequenced on the Illumina NovaSeq 6000 platform (150 bp paired-end). The raw data were quality-filtered using fastp and mapped to the mouse reference genome (GRCm38) using STAR. Gene counts were quantified via featureCounts. Differential expression analysis was performed using DESeq2, while TPM values were calculated for visualization. KEGG pathway enrichment was analyzed using clusterProfiler.

### Single-cell RNA sequencing

Fresh tissues were enzymatically dissociated and filtered to obtain single-cell suspensions. After red blood cell lysis, cell viability was assessed via Countstar. High-quality samples (>80% viability) were processed using the DNBelab C4 system (MGI) for single-cell library preparation following the manufacturer’s protocol. Library quality was verified using Qsep400. The raw sequencing data were processed via the DNBC4 tools pipeline and mapped to the mouse reference genome (GRCm38) using STAR. Downstream analysis was performed using Seurat. Cells with < 500 or > 6,000 detected genes or > 5% mitochondrial content were excluded. The data were normalized, and batch effects were corrected using Harmony. Cell types were annotated via Sc-Type.^26^ In silico knockout analysis was performed using scTenifoldKnk.^27^

### Untargeted metabolomics analysis

The treatment method is consistent with that previously reported.^28^ Briefly, fecal samples (100  mg) were mixed with 1  ml of extraction solvent (methanol:acetonitrile:water, 2:2:1, v/v/v) and ground. After the liquid nitrogen freezing cycle, the cells were sonicated for 10  min. After resting the sample at -20 °C for 1  h, the supernatant was collected by centrifugation, dried under vacuum, and reconstituted in 150  μL of acetonitrile:water (1:1, v/v). Chromatographic separation was performed on a UHPLC system (Ultimate 3000, Thermo Scientific) equipped with an ACQUITY UPLC HSS T3 column (2.1 × 100 mm, 1.8 μm; Waters). The system was coupled to a hybrid quadrupole-orbitrap mass spectrometer (QExactive, Thermo Scientific). Data acquisition was carried out in positive and negative ionization modes using Full MS and ddMS.

### Lipidomics analysis

Liver samples (100 mg) or cell samples were homogenized in 3 mL of dichloromethane:methanol (2:1, v/v) and subsequently subjected to sonication for 30 min. Following the addition of 1 mL of ultrapure water, the sample was vortexed and centrifuged, and the lower organic layer was carefully collected. The residue was re-extracted with an additional 2.0 mL of dichloromethane:methanol (2:1, v/v). After centrifugation, the lower organic layers from both extractions were combined and evaporated to dryness under a gentle stream of nitrogen. The dried residue was then reconstituted in 300 μL of dichloromethane:methanol (1:1, v/v) containing 5 mM ammonium formate for LC‒MS analysis. The instrument conditions refer to untargeted metabolomics, and the elution method can be found in Table S3.

### Targeted metabolomics analysis

Portal vein blood, liver, and fecal samples were prepared and analyzed following the procedures described previously.^25^ Briefly, samples were homogenized in a 0.1% formic acid solution, flash-frozen in liquid nitrogen, and subsequently thawed on ice. The samples were then incubated in a water bath at 75 °C. Following ultrasonic extraction, the mixtures were centrifuged, and the resulting supernatants were mixed with internal standards for detection. Chromatographic separation and detection were carried out using a Sciex 5500 Qtrap mass spectrometer (Sciex, USA) with LC-20A liquid chromatography (Shimadzu, Japan). For targeted metabolomics-related mobile phases and elution methods, please consult Tables S4 and S5.

### Multiplex immunofluorescence staining

Formalin-fixed paraffin-embedded (FFPE) sections were incubated at 4 °C overnight with the following primary antibodies: anti-Tomm20 antibody (1:10 000), anti-CD36 antibody (1:200), anti-PPARγ antibody (1:6000), and anti-ATP1A1 antibody (1:10 000). After washing, the sections were incubated at room temperature for 1 h with the following tyramide-conjugated secondary antibodies: iF440-Tyramide labeled anti-rabbit IgG (1:500), iF488-Tyramide labeled anti-rabbit IgG (1:500), iF555-Tyramide labeled anti-rabbit IgG (1:500), iF647-Tyramide labeled anti-rabbit IgG (1:500), and iF750-Tyramide labeled anti-rabbit IgG (1:500). Nuclei were counterstained with DAPI (G1012, Servicebio, 1:500). Images were acquired on a P250 FLASH scanner and spectrally unmixed using CaseViewer software (3DHISTECH).

### Acyl-polyethylene glycol (PEG) exchange assay (APE assay)

The 200  μg of total tissue/cell protein was used as the starting material, after washing with PBS three times to remove serum proteins, resuspended in ice-cold lysis buffer, and sonicated briefly until the viscosity disappeared to achieve complete lysis. Add EDTA to a final concentration of 5 mM, determine the protein concentration by BCA assay, and adjust it to 2 mg/mL. Under room temperature shaking conditions, treat the sample with TCEP (final concentration of 10 mM) for 30 min, then add NEM (final concentration of 25 mM) to block reduced cysteines and incubate with shaking for 2 h. Terminate the reaction by methanol–chloroform–water precipitation, wash twice with methanol, and dry the precipitate under vacuum. Resuspend the precipitate and divide into two groups: one group is added with NH₂OH (final concentration of 0.75 M) to cleave thioesters, and the other group is added with buffer as a control. Incubate with shaking at room temperature for 1 h and then precipitate again. Resuspend the precipitate, add mPEG-mal (final concentration of 1 mM), and incubate for 2 h. After the final precipitation, resuspend in 1 ×  Laemmli buffer, heat at 95 °C for 5 min. The proteins were separated by SDS‒PAGE, and western blotting with an anti-CD36 antibody was conducted to detect the PEGylated and total CD36 bands. Densitometric analysis using ImageJ software was used to quantify the relative palmitoylation level of CD36, with the same calculation method as for the cell samples.

### Human population cohort validation

This research project involving the collection of blood samples adheres to the ethical principles established by the Medical Ethics Committee of Affiliated Hospital of Qingdao University (QYFY WZLL 27720) and conforms to the ethical guidelines of the 1975 Declaration of Helsinki. The 85 subjects were enrolled, including 26 healthy controls, 29 mild MASLD patients, and 30 moderate-severe MASLD patients, with strict grouping criteria and detailed collection of clinical baseline data (Table S6). Fasting venous blood (5 mL) was collected, centrifuged to separate the serum, aliquoted, and stored at −80 °C. Targeted 2HPAA detection was performed by LC–MS/MS.

### Statistical analyses

Statistical analyses were conducted using GraphPad Prism (v10.1.2) and R (v4.4.3). Data are presented as mean ± standard error of the mean (SEM). Student's t-test was applied for comparisons between two groups, while the Mann‒Whitney U test was used for non-normally distributed data. For comparisons involving multiple groups, one-way ANOVA was utilized. A *P*-value < 0.05 was considered statistically significant.

## Results

### *B. cellulosilyticus* deficiency in MASLD patients

**Figure 1.**
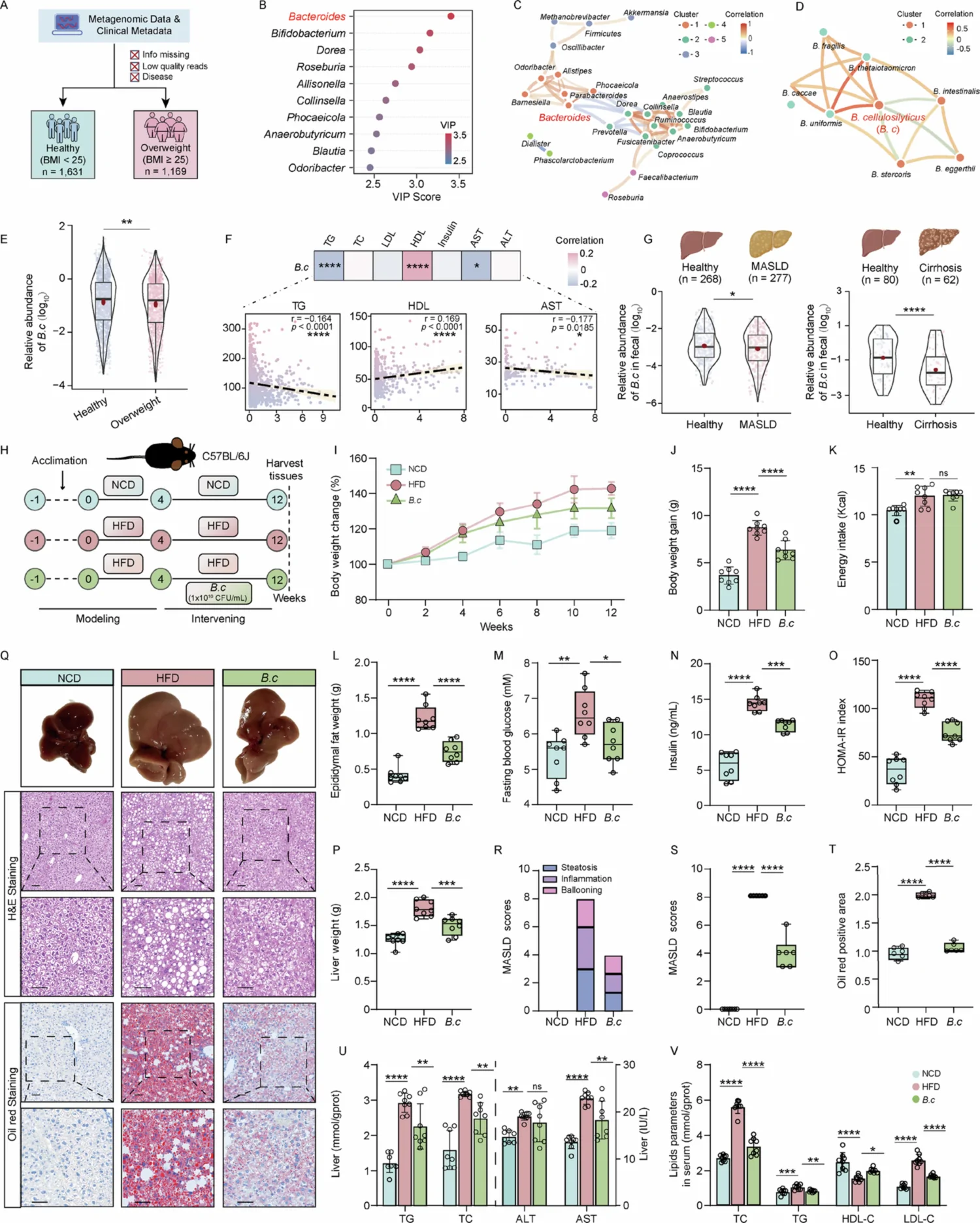
Depletion of *B. cellulosilyticus* correlates with metabolic disorders, and its supplementation alleviates MASLD in mice. (A) Schematic of the study design and metagenomic workflow. (B) PLS-DA VIP scores. (C) Co-occurrence networks of core genera in healthy and overweight subjects. (D) Co-occurrence networks of *Bacteroides* species in healthy and overweight subjects. (E) Relative abundance of *B. cellulosilyticus* in healthy and overweight groups. (F) Correlations between *B. cellulosilyticus* and serum indices. (G) Relative abundance of *B. cellulosilyticus* in the MASLD and cirrhosis cohorts. (H) Experimental design schematic. (I) Body weight changes of mice during the experimental period. (J) Final body weight gain. (K) Energy intake. (L) Epididymal fat weight. (M) Fasting blood glucose levels. (N) Fasting insulin levels. (O) HOMA-IR index. (P) Liver weight. (Q) Representative images of livers, hepatic H&E staining, and Oil Red O staining (scale bar = 50 μm). MASLD scoring (R) and statistical analysis (S). (T) Quantitative analysis of Oil Red O-positive lipid areas. (U) Hepatic levels of TC, TG, ALT, and AST. (V) Serum levels of TC, TG, HDL-C, and LDL-C. Data presented as mean ± SEM. Compared with the indicated groups, *P < 0.05, **P < 0.01, ***P < 0.001, ****P < 0.0001, ns: no significant.

To explore potential gut microbiota candidates associated with metabolic alterations in MASLD, a large-scale human cohort consisting of 1,631 healthy (BMI < 25) and 1,169 overweight (BMI > 25) individuals from curatedMetagenomicData was used as the discovery cohort (Figure 1A and Figure S1A–C). VIP and co-occurrence network analysis indicated that *Bacteroides* was a prominent differential genus, occupying a central topological position in the microbial ecological network (Figure 1B, C and Figure S1D). Further co-occurrence network analysis of *Bacteroides* genus identified *B. cellulosilyticus* as the key species (Figure 1D), with significantly lower relative abundance in overweight individuals (Figure 1E).

Furthermore, among subjects with available biochemical metadata, the abundance of *B. cellulosilyticus* showed a significant negative correlation with serum TG levels and serum AST activity, alongside a significant positive correlation with HDL-C levels (Figure 1F). Subsequently, we evaluated *B. cellulosilyticus* abundance in two independent clinical cohorts.^29^ ^,^ ^30^ The results revealed a consistent reduction of this bacterium in patients with MASLD and cirrhosis compared to healthy controls. *B. cellulosilyticus* was also less abundant in patients with T2D and IGT (Figure 1G and Figure S1E). Together, these large-cohort observations provide preliminary ecological clues that *B. cellulosilyticus* deficiency might be linked to MASLD-related metabolic disorders, warranting functional validation as a potential probiotic candidate.

### *B. cellulosilyticus* supplementation alleviates MASLD in mice

To test whether *B. cellulosilyticus* treatment prevents MASLD in mice, we used *B. cellulosilyticus* AF17-25, which was isolated and purified by BGI from the human gut, and conducted animal experiments on HFD-induced MASLD mice (Figure 1H). After 8 weeks of supplementation intervention, *B. cellulosilyticus* successfully colonized the mice (Figure S2A). The body weight of the mice (Figure 1I–J) significantly decreased, and this decrease was not caused by differences in dietary calorie intake (Figure 1K) or food intake (Figure S2B). Meanwhile, *B. cellulosilyticus* supplementation reduced epididymal fat mass (Figure 1L), fasting blood glucose (Figure 1M), fasting insulin (Figure 1N), and HOMA-IR (Figure 1O), indicating improved insulin sensitivity (Figure S2C, D). It also decreased liver weight versus the HFD group (Figure 1P). Further H&E and Oil Red O staining of liver tissues showed that *B. cellulosilyticus* supplementation reduced lipid droplet accumulation and improved tissue morphology, alleviating hepatic steatosis (Figure 1Q–T and Figure S2E). Biochemical analysis revealed significantly lower hepatic TG, TC, and AST levels (Figure 1U). The serum TC and TG levels also decreased, while HDL-C level increased and LDL-C level decreased (Figure 1V). These results indicate that supplementation with *B. cellulosilyticus* can improve insulin sensitivity, alleviate hepatic steatosis, and regulate lipid metabolism in mice, thereby exerting a protective effect against MASLD.

### 2HPAA is the key metabolite responsible for *B. cellulosilyticus*

**Figure 2.**
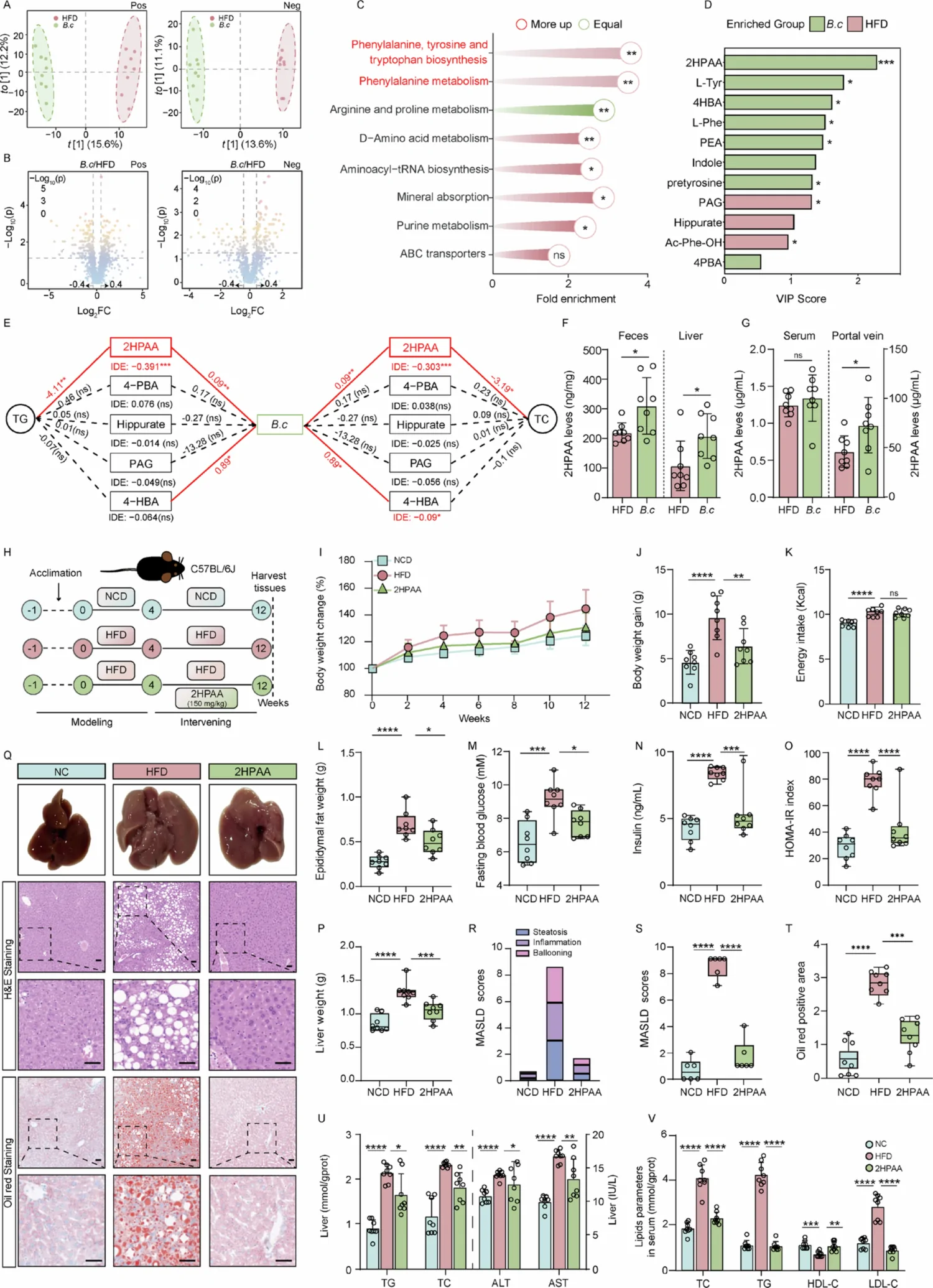
2HPAA serves as the key protective mediator in alleviating MASLD. (A) OPLS-DA of fecal untargeted metabolomics (negative ion mode and positive ion mode). (B) Volcano plots identifying differential metabolites. (C) KEGG pathway enrichment analysis. (D) Bar plot of metabolites involved in the significantly enriched pathways. (E) Mediation analysis of phenylalanine metabolites on TG and TC levels. Targeted metabolomic analysis of 2HPAA levels in the feces, liver (F), and portal vein (G). (H) Experimental design schematic. (I) Body weight changes of mice during the experimental period. (J) Final body weight gain. (K) Energy intake. (L) Epididymal fat weight. (M) Fasting blood glucose levels. (N) Fasting insulin levels. (O) HOMA-IR index. (P) Liver weight. (Q) Representative images of livers, hepatic H&E staining, and Oil Red O staining (scale bar = 50 μm). MASLD scoring (R) and statistical analysis (S). (T) Quantitative analysis of Oil Red O-positive lipid areas. (U) Hepatic levels of TC, TG, ALT, and AST. (V) Serum levels of TC, TG, HDL-C, and LDL-C. Data are presented as mean ± SEM. Compared with the indicated groups, *P < 0.05, **P < 0.01, ***P < 0.001, ****P < 0.0001, ns: no significant.

To identify the key metabolites that mediate the protective effect of *B. cellulosilyticus* against MASLD, we performed metabolomic analysis of fecal, liver, and portal vein samples from the MASLD mouse model. Orthogonal projections to latent structures discriminant analysis (OPLS-DA) and differential analysis based on non-targeted fecal metabolomics indicated that *B. cellulosilyticus* treatment significantly reshaped the fecal metabolic profile (Figure 2A, B and Figure S3A, B). KEGG pathway enrichment analysis indicated that the biosynthesis of phenylalanine, tyrosine, and tryptophan, as well as phenylalanine metabolism, were the most significantly enriched pathways (Figure 2C). We further verified that aromatic amino acid metabolites, especially 2HPAA, exhibited differential abundance (Figure 2D). Subsequently, a mediation analysis of five candidate metabolites identified 2HPAA as the key effector molecule linking the gut microbiota to biochemical phenotypes, such as TC and TG (Figure 2E and Figure S3C, D). We further developed a targeted detection method for 2HPAA and observed that after the treatment with *B. cellulosilyticus*, the concentration of 2HPAA in feces, portal vein, and liver all exhibited a significant increase (Figure 2F-G).

### Exogenous supplementation of 2HPAA alleviates MASLD in mice

Based on our preliminary dose‒response assessment, we identified that 150 mg/kg was the optimal treatment dose (Figure S4). To determine whether 2HPAA can alleviate MASLD directly, we conducted animal experiments (Figure 2H), along with transcriptomic and lipidomic analyses of liver tissues (Figure S5). Exogenous supplementation of 2HPAA altered body weight dynamics (Figure 2I), reduced body weight gain (Figure 2J), without a change in energy intake (Figure 2K), excluding dietary interference with its effects. In addition, 2HPAA effectively reduced the epididymal fat weight (Figure 2L) and improved the systemic metabolic status: the fasting blood glucose level (Figure 2M), insulin level (Figure 2N), and HOMA-IR index (Figure 2O) were all decreased, indicative of ameliorated insulin resistance (Figure S6A, B). Furthermore, 2HPAA reduced the liver weight (Figure 2P). Histological analysis showed that HFD-induced hepatic steatosis and lipid droplet accumulation were significantly alleviated in the 2HPAA group (Figure 2Q). Quantitative evaluation confirmed the decrease in MASLD scores (Figure 2R, S, Figure S6C) and the reduction in Oil Red O-positive lipid areas (Figure 2T). Biochemical tests verified that 2HPAA reduced hepatic TC, TG, ALT and AST levels (Figure 2U), while normalizing the serum lipid profile (TC, TG, HDL-C, and LDL-C; Figure 2V). Transcriptome analysis revealed that differentially expressed genes (DEGs) were significantly enriched in lipid-related biological processes. Consistently, RT-PCR confirmed the significant upregulation of the fatty acid oxidation gene Cpt1a, whereas genes involved in lipid transport and adipogenesis, including *Mttp*, *Pparg2*, *Apob*, and *Cd36*, were markedly downregulated (Figure S5A‒G). Lipidomics showed that *B. cellulosilyticus* intervention markedly reduced hepatic triglyceride accumulation (Figure S5H, I), indicating that the core protective effect of 2HPAA lies in maintaining hepatic lipid homeostasis.

### Causal role of 2-HPAA and *B. cellulosilyticus* in anti-MASLD

**Figure 3.**
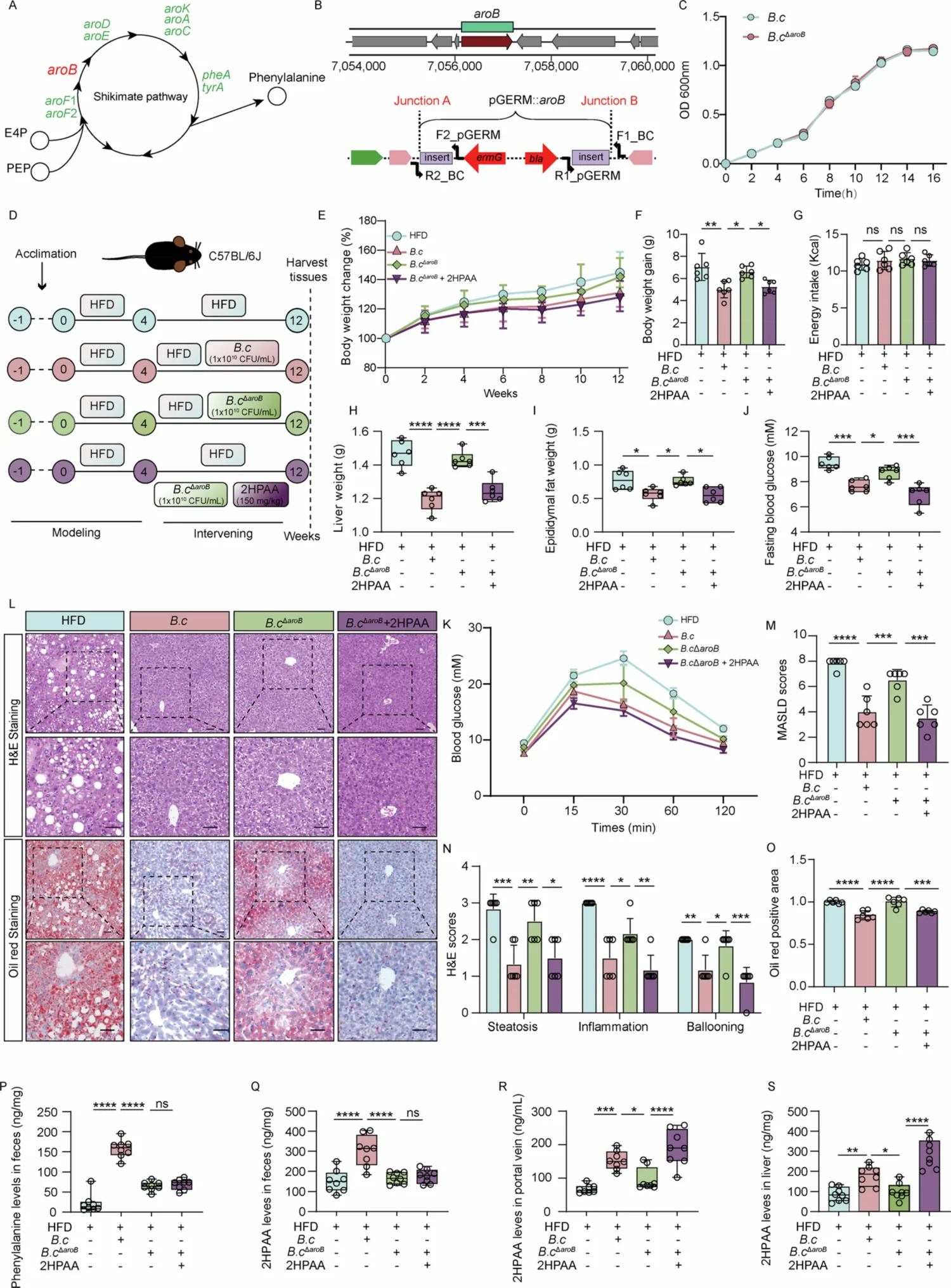
*B. cellulosilyticus* ^*∆aroB*^ impairs the MASLD-alleviating effect. (A) Shikimate pathway for phenylalanine biosynthesis in *B. cellulosilyticus*. (B) Mutant construction. (C) Growth of *B.c* ^Δ*aroB*^ and *B.c* on MYPG medium. (D) Experimental design schematic. (E) Body weight changes of mice during the experimental period. (F) Final body weight gain. (G) Energy intake of the mice. (H) Liver weight of the mice. (I) Mass of epididymal fat tissue. (J) Fasting blood glucose levels. (K) OGTT blood glucose curves. (L) Representative images of livers, hepatic H&E staining, and Oil Red O staining (scale bar = 50 μm). (M) Statistical analysis of MASLD histological scores. (N) MASLD histological scoring criteria (steatosis, inflammation, ballooning). (O) Quantitative analysis of Oil Red O-positive lipid areas. (P) Phenylalanine levels in feces. 2HPAA levels in feces (Q), portal vein (R), and liver (S). Data are presented as the mean ± SEM. *P < 0.05, **P < 0.01, ***P < 0.001; ns, no significance.

Our genome-wide analysis demonstrated that *B. cellulosilyticus* has a robust carbohydrate metabolic system, which provides initial substrates for the shikimate pathway to synthesize phenylalanine (Figure 3A, Figure S7A–C). As expected, targeted amino acid detection confirmed that *B. cellulosilyticus* supplementation significantly increases the contents of phenylalanine, which serve as the precursors for downstream 2HPAA production (Figure S7D). In the shikimate pathway, the 3-dehydroquinic acid synthase encoded by the *aroB* gene acts as the key rate-limiting enzyme.^31^ To confirm the causal role of *B. cellulosilyticus* in 2HPAA-mediated anti-MASLD effects, we generated an *aroB* gene knockout strain of *B. cellulosilyticus* (*B. c* ^*∆aroB*^) (Figure 3B and Figure S8A, B). Growth was unaffected by *aroB* deletion (Figure 3C).

Multi-dimensional phenotypic analysis in the HFD-induced MASLD animal model (Figure 3D) indicated that 2HPAA mediates the weight-reducing effect of *B. cellulosilyticus* via the *aroB* gene. Specifically, *B. c* ^∆*aroB*^ lost this effect, but regained it upon supplementation with 2HPAA (Figure 3E, F). Energy intake remained constant among all groups (Figure 3G). Furthermore, liver phenotype analysis revealed elevated liver and WAT weights in the *B. c* ^∆*aroB*^ group, which were reversed by 2HPAA (Figure 3H–I). The mutant of the *aroB* gene also displayed higher fasting glucose and impaired glucose tolerance, both of which were restored by 2HPAA (Figure 3J, K). In addition, H&E scoring and Oil Red O staining quantification confirmed the aggravation of hepatic steatosis in mice treated with *B. c* ^∆*aroB*^, and this was ameliorated by 2HPAA supplement (Figure 3L–O). Our targeted metabolomics showed *aroB* deletion abolished fecal phenylalanine and 2HPAA over-production (Figure 3P, Q). The synchronously reduced 2HPAA levels in the portal vein and liver of the *B. c* ^∆*aroB*^ group (Figure 3R, S) further support that 2HPAA derived from *B. cellulosilyticus* can be absorbed into the portal circulation and delivered to the liver to exert an anti-MASLD effect.

### 2HPAA alleviates MASLD by inhibiting the hepatic PPARγ signaling pathway

**Figure 4.**
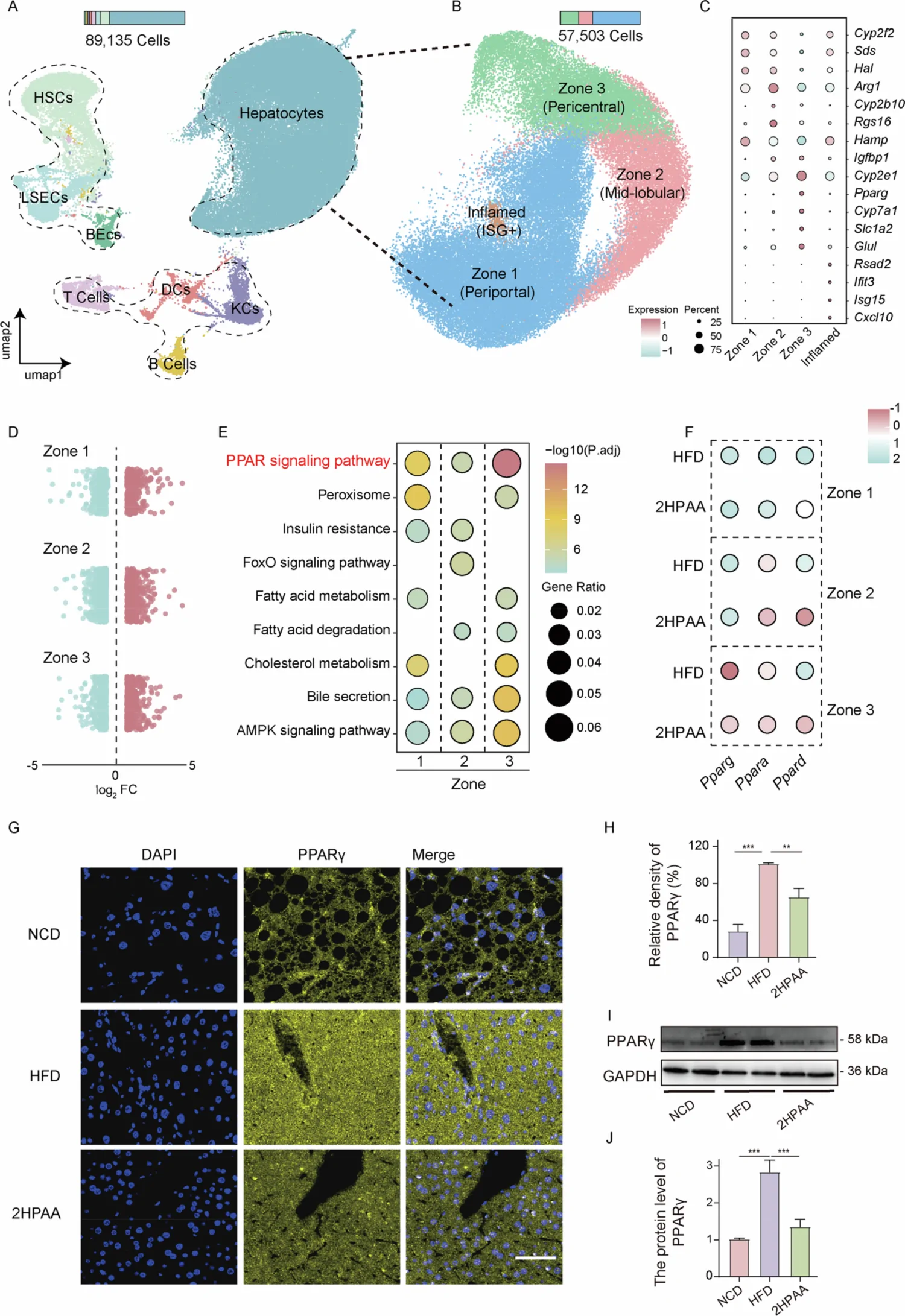
Hepatic PPARγ activation is inhibited by 2HPAA to alleviate MASLD. (A) UMAP plot of the liver cell atlas. (B) UMAP re-clustering of hepatocyte subpopulations. (C) The expression patterns of canonical zonation marker genes across different hepatocyte subpopulations. (D) Distribution of DEGs across hepatocyte zones after 2HPAA treatment. (E) KEGG pathway enrichment of DEGs. (F) Heatmap of PPAR subtype expression across zones. (G) Representative immunofluorescence image of hepatic PPARγ. (scale bar = 50 μm). (H) Quantification of PPARγ fluorescence intensity. (I) Western blot analysis of hepatic PPARγ expression. (J) Quantitative analysis of PPARγ protein levels. Data are presented as mean ± SEM. Compared with the indicated group, ***P < 0.001.

To investigate how 2HPAA alleviates MASLD, we performed single-cell transcriptomic sequencing on liver tissue (Figure 4A). Following rigorous quality control, 89,135 high-quality cells were retained and initially clustered into 13 subpopulations (Figure S9A). Combined annotation via classical cell markers (Figure S9B) and the ScType tool (Figure S9C) classified these cells into 8 major cell types, including hepatocytes, hepatic stellate cells, Kupffer cells, liver sinusoidal endothelial cells, T cells, dendritic cells, cholangiocytes, and B cells (Figure 4A and Figure S9D). Given the central role of hepatocytes in the progression of MASLD,^32^ we focused on how 2HPAA regulates them. Firstly, we obtained 57,503 high-quality hepatocytes, which accounted for 64% of the total cells. Then, re-clustering using liver zonation markers (Figure 4C and Figure S10) revealed three spatial subpopulations (Zones 1–3) and a minor interferon-stimulated gene (ISG)-high subpopulation (0.6%, excluded from downstream analysis) (Figure 4B). In addition, differential expression analysis of zonated hepatocytes (|log₂FC|>0.25, p.adj < 0.05) identified 927, 1,480, and 842 DEGs in Zones 1, 2, and 3, respectively (Figure 4D).

Furthermore, functional enrichment analysis (Figure S11A–C) indicated a significant enrichment of the PPAR signaling pathway throughout Zone 3 (pericentral) (Figure 4E). More importantly, the pericentral zone of hepatocytes is the hypoxic core for *de novo* lipogenesis and the primary site of MASLD.^33^ To clarify the key PPAR subtype mediating these effects, we analyzed *Ppara*, *Ppard*, and *Pparg* expression in zonated hepatocytes, and found that 2HPAA significantly downregulated *Pparg* in Zone 3 (Figure 4F). Consistent with our analysis, tissue-level analyses using immunofluorescence (Figure 4G, H) and western blot (Figure 4I, J) confirmed that 2HPAA treatment significantly reduces hepatic PPARγ protein expression. While molecular docking suggests a theoretical non-covalent binding mode between 2HPAA and PPARγ (Figure S12), further experimental validation is still needed to determine whether 2HPAA regulates PPARγ *via* direct interaction or indirect upstream signaling.

### PPARγ inhibition reduced CD36 palmitoylation

**Figure 5.**
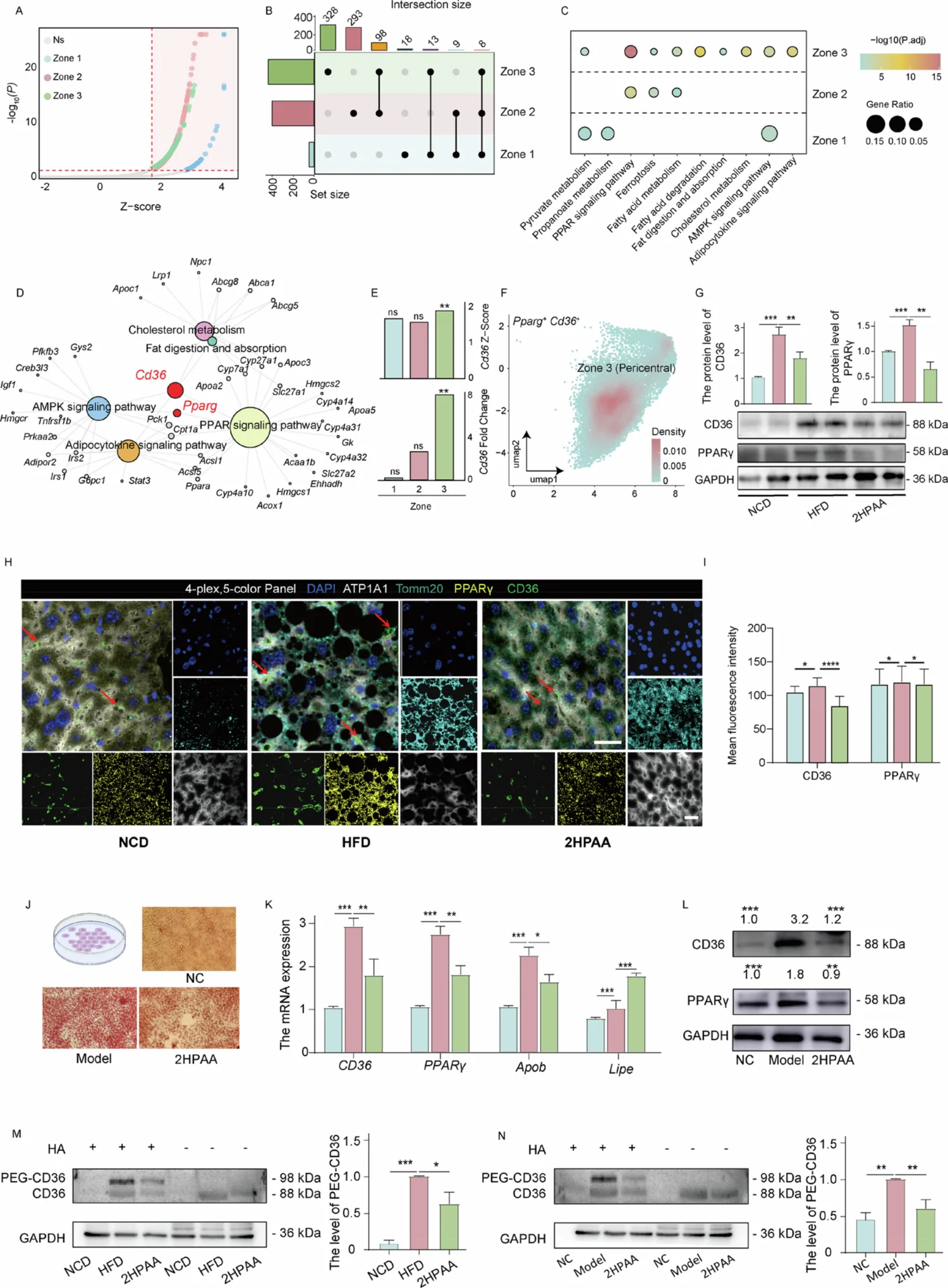
Dual regulation of CD36 is the key mechanism of 2HPAA. (A) In silico perturbation analysis using *Pparg* knockout. (B) UpSet plot of perturbed gene overlap across zones. (C) Functional enrichment of zone-specific perturbed genes. (D) *Pparg* gene regulatory network in Zone 3. (E) Changes in *Cd36* expression following simulated *Pparg* knockout. (F) Co-expression density of *Pparg* and *Cd36* in Zone 3. (G) Hepatic CD36 and PPARγ protein levels. (H) Multiplex immunofluorescence of subcellular localization (scale bar = 50 μm). (I) Quantification of CD36 and PPARγ fluorescence. (J) Representative images of Oil Red O staining in hepatocytes. (K) mRNA expression levels of *Cd36*, *Pparg*, *Apob*, and *Lipe* in hepatocytes. (L) Hepatocyte CD36 and PPARγ protein levels. (M) Hepatic CD36 palmitoylation levels. (N) Hepatocyte CD36 palmitoylation levels. Data are presented as mean ± SEM. Compared with the indicated groups, *P < 0.05, **P < 0.01, ***P < 0.001, ****P < 0.0001, ns: no significant.

To investigate the downstream regulatory effects mediated by *Pparg*, we performed in silico knockout and perturbation analysis at the single-cell level. Based on the significance criteria (Z-score > 1.645, *p* < 0.05), we identified 48, 408, and 447 perturbed genes in Zones 1–3, respectively (Figure 5 A). The overlap and zone-specific distribution of these genes are visualized in Figure 5B. Enrichment analysis of zone-specific perturbed genes revealed that Zone 3 perturbations were primarily concentrated in lipid metabolism, uptake, and transport pathways (Figure 5C), where the exogenous fatty acid transporter *Cd36* was a key hub gene (Figure 5D). *Pparg* deletion-induced *Cd36* perturbation was most pronounced in Zone 3 (Figure 5E), and joint density analysis confirmed a distinct *Pparg* ^+^/*Cd36* ^+^ cell population in this zone (Figure 5F), demonstrating a tight coupling between *Pparg* (PPARγ) activity and Cd36-mediated fatty acid uptake.

To verify the regulatory effects of 2HPAA on the PPARγ/CD36 axis, we conducted experiments spanning tissue localization, molecular expression, and functional modification. As shown in Figure 5G, 2HPAA treatment markedly reduced the protein levels of PPARγ/CD36. The 4-plex immunofluorescence staining revealed significantly lower fluorescence intensities of CD36 and PPARγ after 2HPAA treatment, indicating specific inhibition of PPARγ/CD36 expression in hepatocytes (Figure 5H, I).

In the HepG2 cell lines, lipid droplet staining revealed substantial aggregation in the model group, which was mitigated by 2HPAA treatment (Figure 5J). We also found that the expression of CD36 and PPARγ was elevated at both the mRNA level (Figure 5K) and the protein level (Figure 5L). PEG-labeled palmitoylation assays conducted on both liver tissue (Figure 5M) and cell lines (Figure 5N) showed that 2HPAA reduces CD36 palmitoylation, inhibits PPARγ, subsequently decreases fatty acid uptake, and restores lipid homeostasis.

### 2HPAA reduces hepatic lipid accumulation by inhibiting PPARγ

**Figure 6.**
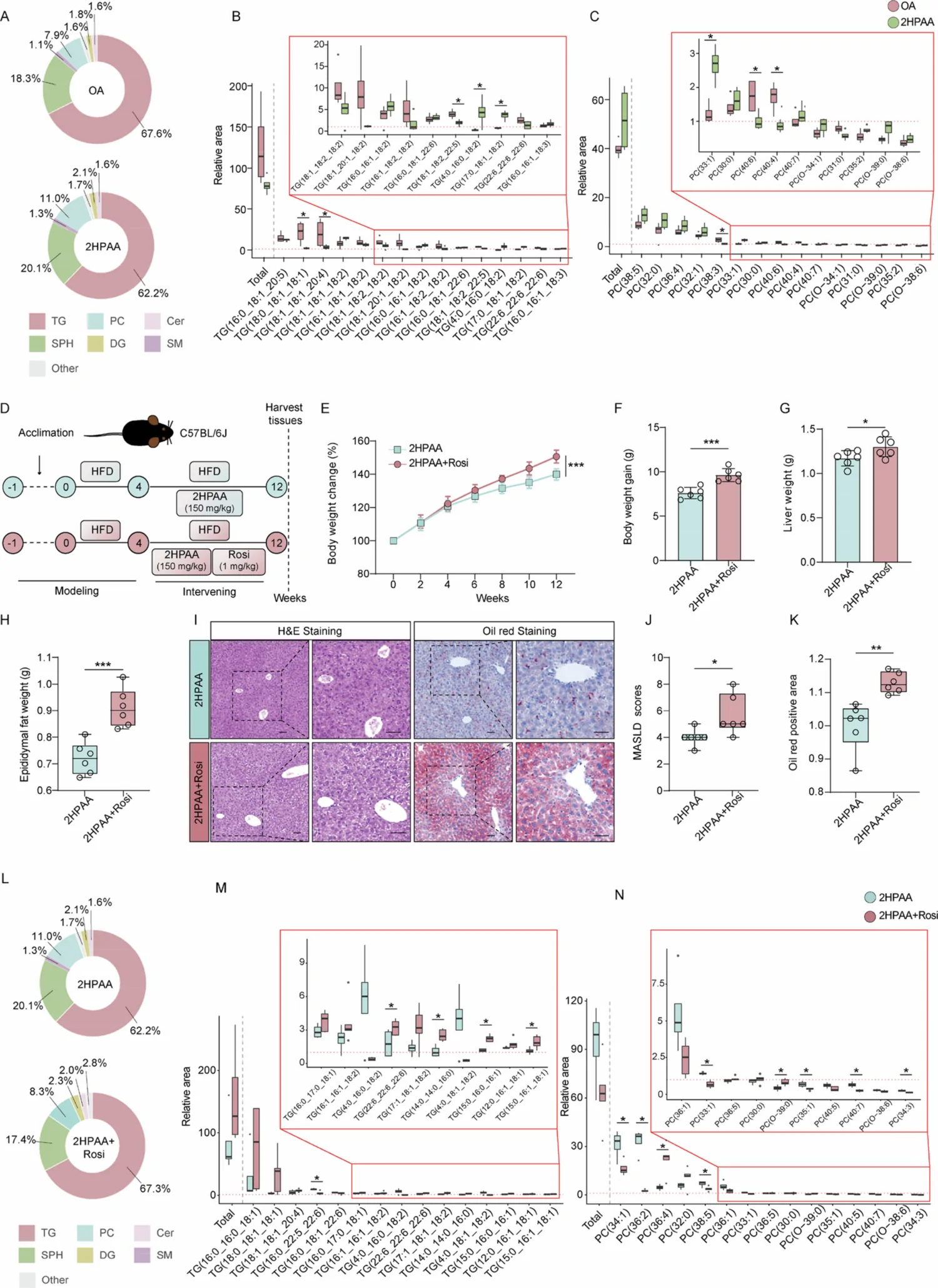
2HPAA alleviates MASLD via PPARγ-dependent regulation of lipid homeostasis. (A) Lipid subclass composition in the OA model group and the 2HPAA treatment group. Total amounts and relative abundance of specific TG (B) and PC (C) species. (D) Experimental design schematic. (E) Body weight changes of mice during the experimental period. (F) Final body weight gain. (G) Liver weight. (H) Mass of epididymal fat tissue. (I) Representative images of livers, hepatic H&E staining, and Oil Red O staining (scale bar = 50 μm). (J) Statistical analysis of MASLD histological scores. (K) Quantitative analysis of Oil Red O-positive lipid areas. (L) Lipid subclass composition in the 2HPAA group and the 2HPAA + Rosi group. Total amounts and relative abundance of specific TG (M) and PC (N) species. Data presented as mean ± SEM. Compared with the indicated groups, *P < 0.05, **P < 0.01, ***P < 0.001, ****P < 0.0001, ns: no significant.

We employed lipid metabolomics to evaluate the beneficial effect of 2HPAA on lipid homeostasis in HepG2 cell lines (Figure 6A‒C) and liver tissue (Figure S13). Treatment with 2HPAA significantly reshaped the overall lipid composition (Figure 6A). Total TG accumulation and specific high-VIP TG species were significantly reduced in the 2HPAA group (Figure 6B), whereas the proportion and abundance of PC were increased (Figure 6C). Next, we confirmed the critical role of PPARγ in 2HPAA-mediated MASLD alleviation through a 12-week HFD-fed animal experiment using the PPARγ agonist, Rosiglitazone (Figure 6D). As expected, the Rosiglitazone-treated group significantly increased body weight gain (Figure 6E, F), liver and epididymal fat weight (Figure 6G, H), reversed 2HPAA’s improvement in hepatic steatosis, and raised MASLD scores and Oil Red O-positive areas (Figure 6I–K). Further analysis of the overall lipid composition confirmed that the favorable lipid profile induced by 2HPAA was reversed in the Rosiglitazone group (Figure 6L). Quantitative profiling further revealed that the Rosiglitazone group showed a significant increase in total TG accumulation (Figure 6M) and a decrease in total PC levels (Figure 6N). These results imply that Rosiglitazone weakens the inhibitory effect of 2HPAA on PPARγ, thus impairing the ability of 2HPAA to regulate lipid homeostasis.

### Population cohort validation of 2HPAA and MASLD progression

**Figure 7.**
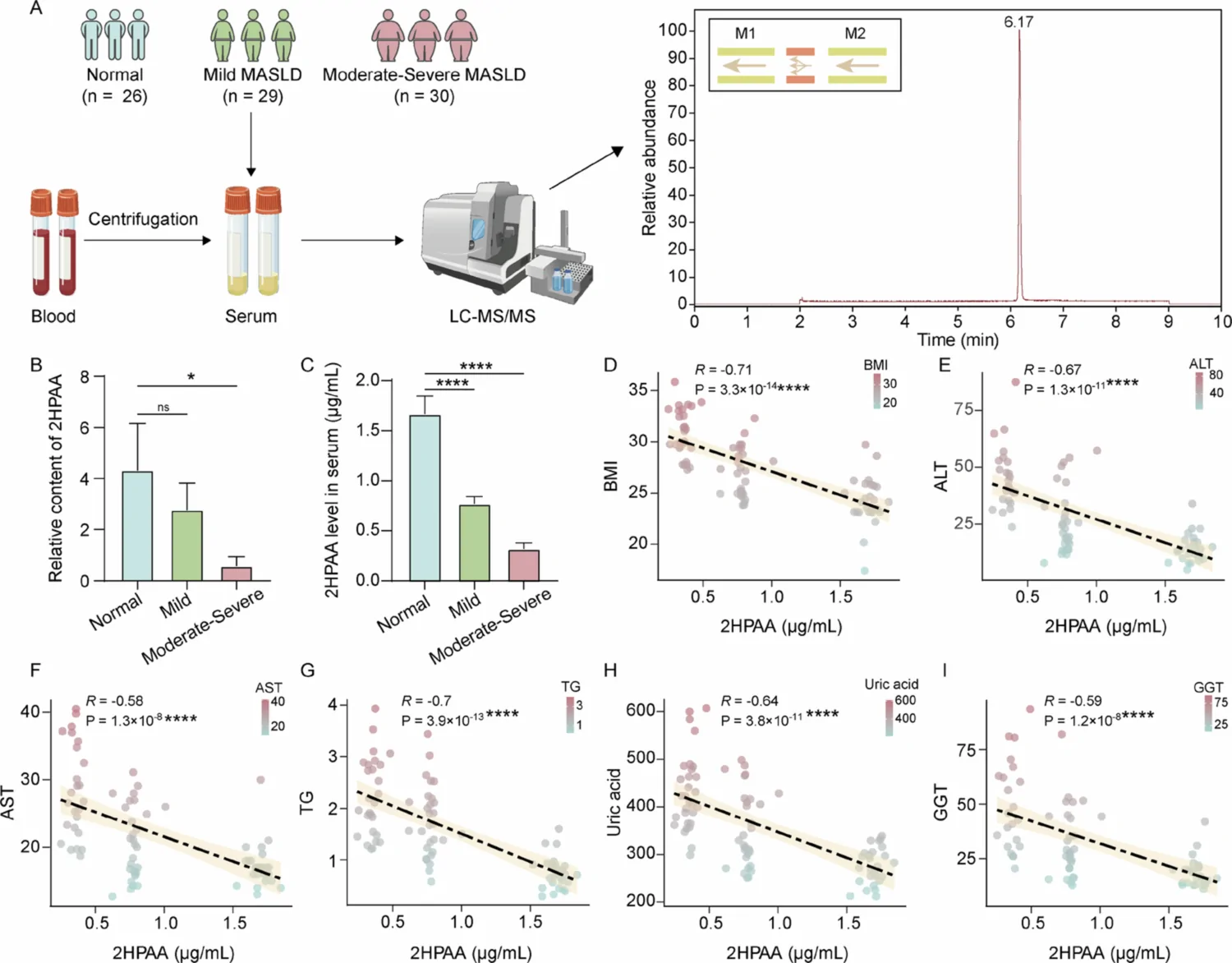
Population cohort validation of the correlation between 2HPAA and MASLD. (A) Analysis process of the population cohort (*n* = 26 for normal, *n* = 29 for mild MASLD, and *n* = 30 for moderate-severe MASLD). (B) Relative content of 2HPAA in the serum. (C) Absolute quantification of 2HPAA levels in the serum. Correlation of BMI (D), ALT (E), AST (F), TG (G), uric acid (H), and GGT (I) with 2HPAA levels. Data are presented as mean ± SEM. Compared with the indicated groups, *P < 0.05, ****P < 0.0001; ns, no significance.

To explore the clinical relevance of 2HPAA and MASLD, serum samples were collected from healthy individuals and patients with varying degrees of MASLD for metabolomics analysis (Figure 7A). Both the results of non-targeted metabolomics and targeted metabolomics indicated that the serum 2HPAA content exhibited a significant downward trend as the severity of MASLD increased (Figure 7B, C). Further correlation analysis demonstrated a close link between the serum 2HPAA level and the body's metabolic health. The serum 2HPAA concentration showed a significant negative correlation with BMI, indicators reflecting liver injury (ALT, AST, and GGT), and metabolic disorders (TG, uric acid) (Figure 7D–I and Figure S14A–F). Interestingly, this metabolite depletion correlates closely with the ecological fragility of its bacterial producer. Our separate longitudinal cohort revealed a significant reduction in the relative abundance of *B. cellulosilyticus* in 42 volunteers after a 30-day enclosed voyage (Figure S15A, B). From the perspective of special medicine, long-term confined environments impose multiple challenges, including limited mobility, a shifted diet, disturbed circadian rhythms, and prolonged physical and psychological stress. Such composite pressures disrupt gut microbial homeostasis and particularly threaten the survival of fiber-dependent commensals such as *B. cellulosilyticus*. This special population under enclosed conditions is prone to intestinal dysbiosis, and the loss of protective bacteria may predispose individuals to metabolic abnormalities associated with hepatic steatosis. Considering the above clinical data, the deficiency of 2HPAA is not only an important metabolic characteristic of MASLD patients but may also play a crucial role in the progression of the disease and the occurrence of related metabolic disorders. The reduced serum 2HPAA in MASLD patients currently represents an observational association and does not establish a causal relationship or direct therapeutic relevance. Further longitudinal and mechanistic studies are warranted to determine whether this deficiency is a driver of disease progression or a consequence of metabolic dysfunction.

## Discussion

MASLD affects approximately 38% of the global population and is associated with the gut microbiota and its metabolites.^34^ However, the underlying mechanisms remain poorly understood.^35^ Recently, many gut microbiota metabolites, such as amino acid derivatives and short-chain fatty acids (SCFAs), have emerged as critical regulators of metabolic homeostasis.^36-38^ Among aromatic amino acid derivatives, the tyrosine metabolites 4HPAA and 3HPAA have been previously reported to exert potential probiotic effects in metabolic regulation.^19^ ^,^ ^23^ Herein, we identified 2HPAA, a novel phenylalanine derivative, as a highly effective anti-MASLD metabolite, and further clarified its action mechanisms through the gut‒liver axis. These findings offer a potential therapeutic target for the clinical intervention of MASLD.

Through a large cohort, we identified *B. cellulosilyticus* as a keystone commensal, and exogenous supplementation of this potential probiotic is a feasible approach to alleviating MASLD in mice (Figure 1). Genome analysis revealed that *B. cellulosilyticus* possesses a functional shikimate pathway for the generation of 2HPAA precursors. However, a key limitation of our study is that the *hppD* gene, previously reported to be essential for converting phenylalanine to 2HPAA, was not detected in its genome (Figures S16 and S17). Our metagenomic detection revealed that *B. cellulosilyticus* modulates the gut microbiota, enriches taxa encoding *hppD*, upregulates genes involved in 2HPAA synthesis, and promotes 2HPAA production through microbial cross-metabolism (Figure S16). In fact, it might also degrade 2HPAA via an unknown mechanism, which necessitates further research.

A core finding of this study is the established definitive causal relationship between *B. cellulosilyticus* and 2HPAA in mediating anti-MASLD effects (Figure 3). This finding aligns with similar studies showing that microbial metabolites are the key mediators of commensal bacteria‒host interactions, such as *Parabacteroides goldsteinii*-derived isobutyric acid in colitis,^28^ *Butyricimonas virosa*-derived thiamine in MASLD^25^ and *Alistipes indistinctu*s-derived hippuric acid in hyperuricemia regulation.^39^ The consistency of this “microbe-metabolite-target” paradigm across metabolic diseases highlights the generality of microbial metabolites as central messengers in host physiology. Herein, by constructing a *B. cellulosilyticus* strain with a knockout of *aroB*, which is a key gene in the shikimate pathway essential for 2HPAA biosynthesis, we verified that *B. cellulosilyticus* exerts its MASLD-protective activity in a 2HPAA-dependent manner.

Mechanistically, we uncovered that 2HPAA exerts its hepatoprotective effects by directly inhibiting the PPARγ/CD36 signaling pathway. Single-cell sequencing and molecular docking analyses demonstrated that 2HPAA binds to key amino acid residues (Tyr327, Cys285, His323, and Ser289) of PPARγ, functioning as a potential PPARγ inhibitor to suppress CD36 expression. This is distinct from the PPARγ-activating effects of hippuric acid in intestinal urate excretion,^39^ emphasizing the context-dependent roles of microbial metabolites in targeting nuclear receptors. Furthermore, 2HPAA reduces CD36 palmitoylation, a post-translational modification critical for CD36 membrane localization and fatty acid transport activity.^40^ This dual regulation of CD36 effectively inhibits hepatocellular lipid uptake and accumulation at both the transcriptional and post-translational levels, highlighting a precise and multifaceted regulatory mechanism of 2HPAA.

The gut‒liver axis serves as a key conduit for microbial metabolites to modulate hepatic function,^41^ ^,^ ^42^ and our study confirms that 2HPAA enters the liver via enterohepatic circulation to exert direct regulatory effects. Lipidomic analysis further revealed that 2HPAA enhances palmitate conversion efficiency, suggesting additional roles in promoting lipid catabolism beyond inhibiting lipid uptake. This finding is consistent with the observation that 2HPAA upregulates lipolysis-related genes and downregulates lipid transport-associated genes in hepatocytes, indicating the bidirectional regulation of lipid metabolism. Such comprehensive modulation of lipid homeostasis distinguishes 2HPAA from single-target therapeutic agents and underscores its potential as a natural metabolic regulator.

Clinical translation of our findings is supported by the negative correlation between circulating 2HPAA levels and clinically conventional detection indicators (BMI, AST, ALT, GGT, and uric acid) in human cohorts. The safety profile of microbial metabolites, such as ursodeoxycholic acid and hippuric acid,^43^ further suggests that 2HPAA could serve as a novel candidate metabolic marker or therapeutic agent for MASLD. However, it should be emphasized that the current findings primarily support its biological role and its association with MASLD phenotypes, rather than establishing it as a definitive diagnostic or prognostic biomarker. The clinical utility of this candidate metabolic marker requires further rigorous validation in independent, clinically characterized cohorts. Future clinical studies must comprehensively assess its diagnostic discrimination, establish standardized clinical thresholds, and evaluate the influence of various confounding factors before it can be translated into clinical practice. Additionally, the restoration of *B. cellulosilyticus* abundance or exogenous 2HPAA supplementation may represent promising microbiota-based interventions.

However, we must clearly acknowledge the limitations of our human cohort analysis. Although the public metagenomic datasets used in our discovery phase provided a large sample size, they lacked detailed clinical metadata. Most notably, the lack of detailed liver histology and the absence of a specific cirrhosis etiology represent key limitations of this study. Furthermore, because detailed dietary metadata was unavailable in these public cohorts, the influence of habitual diet on the colonization and abundance of *B. cellulosilyticus* in humans was not evaluated in the present study. Future research needs to include biopsy-proven MASLD clinical cohorts with comprehensive dietary tracking to further validate our findings.

In addition, microbiome-targeted therapies for MASLD fall into two complementary strategies: replenishing depleted beneficial microbes and suppressing overgrown pathogens. While our work focuses on the therapeutic potential of restoring *B. cellulosilyticus* and its metabolite 2HPAA, reducing pathogenic bacteria remains equally important. Recent reports have confirmed that enrichment of taxa, including *Blautia* products, accelerates MASLD progression via its metabolite 2-oleoylglycerol.^44^ Notably, functional food interventions can deplete such pathogens and concurrently boost beneficial commensals such as *Faecalibacterium prausnitzii*, *Akkermansia muciniphila*, and *Ruminococcus bromii.* ^45^ Accordingly, future combinatorial regimens integrating protective strain supplementation and pathogen inhibition may yield synergistic, comprehensive treatment for MASLD.

In conclusion, this study identifies *B. cellulosilyticus*-derived 2HPAA as a key mediator of gut‒liver crosstalk that alleviates MASLD by dual regulation of CD36 expression and palmitoylation via PPARγ inhibition. These findings reveal a novel mechanism by which the gut microbiota regulates hepatic lipid metabolism and provide new insights into the development of targeted therapies for MASLD. By enhancing *B. cellulosilyticus* colonization, endogenous supplementation of 2HPAA, or targeting PPARγ/CD36 axis may offer a promising strategy for the prevention and treatment of this prevalent metabolic liver disease.

## Supplementary Material

Supplementary Material Supplymentary materials.doc

## Data Availability

The metabolomic data generated in this study have been deposited in CNGBdb with the serial number CNP0008999 (https://db.cngb.org/data_resources/?query=CNP0008999). The scRNA-seq data have been deposited in CNP0009002 (https://db.cngb.org/data_resources/?query=CNP0009002). Non-targeted metabolomic data are publicly accessible via the GitHub repository at https://github.com/illusion621/Bc_2HPAA. Any additional information required to re-analyze the reported data is available from the lead contact upon request.
