# Supplementary material for: Bacteroides cellulosilyticus-derived 2-hydroxyphenylacetic acid rectifies hepatic lipid homeostasis in MASLD by targeting the PPARγ-CD36 axis
Source: Gut Microbes. 2026 Sep 1;18(1):2725392. doi: 10.1080/19490976.2026.2725392 (PMC13540161; doi:10.1080/19490976.2026.2725392)
Supplement: Supplementary file 1 [file KGMI_A_2725392_SM8785.doc]

***Bacteroides cellulosilyticus*-derived 2-hydroxyphenylacetic acid rectifies hepatic lipid homeostasis in MASLD by targeting the PPARγ-CD36 axis**

Kaiwei Chen^1^†, Zizhen Yang^1^†, Jixing Peng^2^†, Chunyan Liu^1^†, Ying Yu^1^, Xinchi Cai^1^, Bei Liu^1^, Shuao Li^1^, Tiantian Chen ^1,2^, Samil Jung ^3^, Yu Tian ^4,5^, Qianyue Xu ^4,6^, Xing Rao ^4,6^, Zhinan Wu ^4,6^, Haoyu Wang ^4,6^, Yan Di ^2^, Linna Wang ^2^, Jianxun Wang ^1^*, Myeong-sok Lee ^3^*, Yuanqiang Zou ^4,7^*, Ningning He ^1^*, Shangyong Li ^1^*

^1^School of Basic Medicine, Qingdao Medical College, Qingdao University, Qingdao 266071, China.

^2^Yellow Sea Fisheries Research Institute, Chinese Academy of Fishery Sciences, Qingdao 266071, China.

^3^Molecular Cancer Biology Laboratory, Cellular Heterogeneity Research Center, Department of Biosystem, Sookmyung Women's University, Seoul 140-742, Korea.

^4^State Key Laboratory of Genome and Multi-omics Technologies, BGI Research, Shenzhen 518083, China.

^5^College of Life Sciences, Northwest University, Xi'an 710069, China.

^6^College of Life Science, University of Chinese Academy of Sciences, Beijing 100049, China.

^7^Shenzhen Key Laboratory of Human commensal microorganisms and Health Research, BGI Research, Shenzhen 518083, China.

† These authors contributed equally to this work.

*Corresponding author:

Jianxun Wang, School of Basic Medicine, Qingdao Medical College, Qingdao University, Qingdao 266071, Email: wangjx@qdu.edu.cn

Myeong-sok Lee, Molecular Cancer Biology Laboratory, Cellular Heterogeneity Research Center, Department of Biosystem, Sookmyung Women's University, Seoul 140-742, Tel: 82-2-710-9418, Fax: 82-2-2077-7257, Email: mslee@sookmyung.ac.kr

Yuanqiang Zou, State Key Laboratory of Genome and Multi-omics Technologies, BGI Research, Shenzhen 518083, Email: zouyuanqiang@genomics.cn

Ningning He, School of Basic Medicine, Qingdao Medical College, Qingdao University, Qingdao 266071, Email: heningning@qdu.edu.cn

Shangyong Li, School of Basic Medicine, Qingdao Medical College, Qingdao University, Qingdao 266071, Email: lisy@qdu.edu.cn.

**Supplemental Materials and Methods**

**Materials**

C57BL/6J mice (male, 6 weeks old, 16-18 g) were obtained from Jinan Pengyue laboratory animal breeding Co., Ltd (Jinan, China). The Elisa kits for alanine amiotransferase (ALT, C009-2-1), aspartate aminotransferase (AST, C010-2-1), insulin assay kit (H203-1-2), triglyceride (TG, A110-1-1) and total cholesterol (TC, A111-1-1) were purchased from Nanjing Jiancheng Bioengineering Research Institute (Nanjing, China). TG assay kit (S03027), TC assay kit (S03042), ALT assay kit (S03030) and AST assay kit (S03040) for serum biochemical analysis were purchased from Rayto Life and Analytical Sciences Co., Ltd (Shenzhen, China). The antibody for CD36 (A28290), PPARγ (A0270), GAPDH (A19056) were purchased from ABclonal Biotechnology Co., Ltd. (Wuhan, China). The antibody for anti-Tomm20 (GB151481)，anti-ATP1A1 antibody (GB112205), iF440-Tyramide labeled anti-rabbit IgG (G1250), iF488-Tyramide labeled anti-rabbit IgG (G1231), iF555-Tyramide labeled anti-rabbit IgG (G1233), iF647-Tyramide labeled anti-rabbit IgG (G1232), and iF750-Tyramide labeled anti-rabbit IgG (G1258), were purchased from Servicebio (Wuhan, China). HepG2 were originally obtained from the American Type Culture Collection (ATCC, Manassas, VA, USA).

**Animal study**

Body weight and energy intake were measured daily. Fecal samples were collected before and after the intervention and were quickly frozen at -80 °C for subsequent analysis. For the oral glucose tolerance test, mice were fasted for 8 h and then given glucose (2 g/kg/body weight) by oral gavage. HOMA-IRwas calculated using the formula: HOMA-IR = (fasting plasma glucose × fasting insulin) / 22.5. In the end of experiment, following a 12-hour fasting period, mice were anesthetized with inhalational isoflurane. Blood samples were collected via orbital sinus bleeding from mice prior to euthanasia and subsequently centrifuged (3000 rpm, 4 °C, 15 min) to isolate serum samples. In accordance with the experimental procedures, mice were euthanized, and the liver as well as epididymal white adipose tissue depots (eWAT) were aseptically excised and precisely weighed. The collected tissues intended for histological examination were fixed in a 4% paraformaldehyde solution, while the remaining tissues were rapidly frozen in liquid nitrogen and subsequently stored at -80 °C for further analysis.

**Genomic analysis**

Gene prediction and preliminary annotation of the *B. cellulosilyticus* AF17-25 genome were performed using Prokka. Functional annotation was further refined using eggNOG-mapper, and the resulting COG classifications were used for metabolic pathway enrichment analysis.

**Agarose gel electrophoresis**

To verify the disruption of the *aroB* gene, PCR analysis was performed using genomic DNA extracted from the antibiotic-resistant colonies. Specific primers flanking the recombination site were designed to amplify the target region. The PCR products were resolved by 1% agarose gel electrophoresis in 1× TAE buffer at 100 V for 30 min. The gel was stained with SerRed (Servicebio, Wuhan, China) and visualized under UV light. GN8K DNA marker (Servicebio, Wuhan, China) was used to estimate the fragment sizes.

**Cell culture and handling**

The human hepatoma cell line HepG2 was cultured in a standard humidified incubator at 37°C and 5% CO₂. When the cells reached 70%-80% confluence, they were detached using 0.25% trypsin-EDTA solution and subcultured at an appropriate split ratio. The culture medium was replaced every 2-3 days. For subsequent in vitro experiments, HepG2 cells were seeded into appropriate multi-well plates, allowed to adhere overnight, and then subjected to further treatments.

**Enzyme-Linked Immunosorbent Assay (ELISA) and Biochemical Analysis**

For blood sample, collected specimens were centrifuged (3000 rpm, 30 min, 4 °C), and the serum was extracted for subsequent detection after centrifugation. For tissue samples, Tissues were mixed with normal saline (mg:μL =1:9), homogenized, and centrifuged for subsequent analysis (3000 rpm, 10 min, 4 °C). The corresponding ELISA kits were used, and the manufacturer’s instructions were strictly followed to quantitatively determine the levels of the measured parameters. An automatic clinical chemistry analyzer was employed to detect serum TC, TG, low-density lipoprotein cholesterol (LDL-c), and high-density lipoprotein cholesterol (HDL-c).

**Western blotting and quantitative Real-Time PCR (qRT-PCR) analysis**

The total protein of liver tissues or cell samples were isolated using pre-chilled RIPA lysis buffer. After centrifugation (12,000 xg, 15 min, 4 °C), the supernatant was collected. After electrophoresis, transfer, and blocking, the membranes were incubated with primary antibodies at 4 ℃ overnight. Thereafter, the membranes were incubated with secondary antibodies for 1 hour at ambient temperature, and the protein signals were visualized by means of the ECL (Servicebio, Wuhan, China).

The total RNA of liver tissues was isolated using TRIzol reagent following the manufacturer’s instructions. The extracted RNA was then transcribed into cDNA (SweScript All-in-One RT SuperMix, Servicebio, Wuhan, China). Gene expression was measured using the 2×Universal Blue SYBR Green qPCR Master Mix (Servicebio, Wuhan, China), with Actb serving as the internal reference gene. The following primers were used for indicated genes as shown in **Table S7**.

**Histopathological Analysis**

Liver tissues were fixed in 4% paraformaldehyde, paraffin-embedded, and sectioned. Following dewaxing, sections were stained with Hematoxylin and Eosin (H&E). Images were captured using a light microscope to evaluate liver tissue morphology, including hepatocellular steatosis, inflammation, and ballooning. The histologic sections were scored by two blinded pathologists. For Oil Red O staining, sections were stained with filtered Oil Red O working solution. Then, differentiated with 60% isopropanol, rinsed with PBS, and counterstained with hematoxylin. After rinsing with tap water, sections were mounted with glycerol gelatin. Lipid droplets were visualized as red-stained areas under a light microscope, and ImageJ software was used for quantitative analysis of lipid accumulation.

***In silico* Knockout and Perturbation Analysis**

*In silico* knockout analysis was performed using scTenifoldKnk. Stable Gene Regulatory Networks (GRNs) were constructed from expression matrices via a subsampling strategy. Virtual knockout was simulated by zeroing outgoing edges of target genes. Perturbation impacts were quantified using manifold alignment, with significance assessed via Chi-square tests.

**Table S1. Diet details**

|  | NCD | HFD |
| --- | --- | --- |
| Carbohydrate (% of energy) | 64.00 | 20.00 |
| Protein (% of energy) | 20.00 | 20.00 |
| Fat (% of energy) | 7.00 | 60.00 |
| Energy (kcal/kg)  Ingredient, g/kg | 4000.00 | 5240.00 |
| Casein (form milk) | 200.00 | 200.00 |
| Corn starch | 397.49 | 0.00 |
| Maltodextrin 10 | 0.00 | 125.00 |
| Sucrose | 100.00 | 68.8 |
| Dextrose | 132.00 | 0.00 |
| Cellulose | 50.00 | 50.00 |
| Soybean oil | 70.00 | 25.00 |
| Mineral mixture | 35.00 | 10.00 |
| Vitamin mixture | 10.00 | 10.00 |
| TBHQ | 0.01 | 0.00 |
| DL -Methionine | 0.00 | 0.00 |
| L -Cystine | 3.00 | 3.00 |
| Calcium carbonate | 0.00 | 5.50 |
| Cholesterol | 0.00 | 0.00 |
| Choline bitartrate | 2.50 | 2.00 |
| Chokeberry powder | 0.00 | 0.00 |
| DiCalcium Phosphate | 0.00 | 13.00 |
| Calcium Carbonate | 0.00 | 5.50 |
| Potassium Citrate | 0.00 | 16.50 |
| Lard | 0.00 | 245.00 |

**Table S2 Public population information.**

| Disease | Accession Number |
| --- | --- |
| T2D | PRJEB1786,PRJEB37249 |
| IGT | PRJEB1786,PRJEB37249 |
| MASLD | PRJNA1246224,PRJNA728908 |
| Cirrhosis | PRJEB6337 |
| T1D | PRJNA231909 |
| asthma | PRJEB9576 |
| migraine | PRJEB9576 |
| ACVD | PRJEB21528 |
| CAD | PRJEB38742 |

**Table S3 Graded elution program for targeted analysis of lipidomics.**

| Time (min) | A* (Acetonitrile/water, v/v = 6:4) % | B* (Isopropanol/Acetonitrile, v/v = 9:1) % |
| --- | --- | --- |
| 0.0 | 80 | 20 |
| 1.0 | 80 | 20 |
| 5.0 | 50 | 50 |
| 23.0 | 0 | 100 |
| 26.0 | 0 | 100 |
| 26.1 | 80 | 20 |
| 30.0 | 80 | 20 |

***** Contains 0.1% formic acid and 10 mMol/L ammonium formate.

**Table S4 Graded elution program for targeted analysis of 2HPAA**

| Time (min) | A (Acetonitrile contains 0.05% formic acid) % | B (5 mM ammonium acetate aqueous solution contains 0.05% formic acid) % |
| --- | --- | --- |
| 0.0 | 10 | 90 |
| 1.0 | 10 | 90 |
| 3.0 | 30 | 70 |
| 5.0 | 90 | 10 |
| 7.0 | 90 | 10 |
| 7.1 | 10 | 90 |
| 10.0 | 10 | 90 |

**Table S5 Graded elution program for targeted analysis of phenylalanine and tyrosine.**

| Time (min) | A (Acetonitrile contains 0.05% formic acid) % | B (Water contains 0.05% formic acid) % |
| --- | --- | --- |
| 0.0 | 2 | 98 |
| 3.0 | 2 | 98 |
| 5.0 | 15 | 85 |
| 7.0 | 90 | 10 |
| 9.0 | 90 | 10 |
| 9.1 | 2 | 98 |
| 12.0 | 2 | 98 |

**Table S6 Cohorts description**

|  | Normal  (n = 26) | Mild  (n = 29) | Moderate-Severe  (n = 30) | P-Value |
| --- | --- | --- | --- | --- |
| Demographic | | | | |
| Age, y (Q1, Q3) | 33.0 (28.3, 37.0) | 37.0 (34.0, 39.0) | 40.0 (33.0, 48.5) | 0.002 |
| Male sex (%) | 3 (11.5) | 18 (62.1) | 26 (86.7) | <0.001 |
| Female sex (%) | 23 (88.5) | 11 (37.9) | 4 (13.3) | <0.001 |
| Clinical | | | | |
| BMI, mean±SD | 24.20 ± 2.40 | 27.02 ± 2.35 | 31.31 ± 2.88 | <0.001 |
| ALT, U/L (Q1, Q3) | 13.5 (10.3, 17.0) | 23.0 (16.3, 32.8) | 46.4 (38.8, 62.8) | <0.001 |
| AST, U/L (Q1, Q3) | 17.0 (16.0, 18.0) | 20.4 (16.3, 25.9) | 29.2 (23.6, 37.7) | <0.001 |
| TG, μmol/L (Q1, Q3) | 0.71 (0.56, 0.90) | 1.67 (1.23, 2.12) | 2.23 (1.61, 2.91) | <0.001 |
| UA, μmol/L (Q1, Q3) | 273.5 (247.3, 301.8) | 329.7 (294.2, 416.4) | 416.5 (368.7, 475.0) | <0.001 |
| GGT, U/L (Q1, Q3) | 16.5 (14.0, 20.8) | 28.0 (20.7, 44.3) | 61.2 (36.6, 108.5) | <0.001 |

**Table S7 Summary of PCR primers**

| **Gene** | **Forward sequence** | **Reverse sequence** |
| --- | --- | --- |
| **Mouse** | | |
| *GAPDH* | TGTAGACCATGTAGTTGAGGTCA | AGGTCGGTGTGAACGGATTTG |
| *Cpt1a* | GGCCTCTGTGGTACACGACAA | CTCAGTGGGAGCGACTCTTCA |
| *Mttp* | CAGCACCTCCGGACTTCGAGGA | TGAGCAGAGGTGACGGCATCCA |
| *Pparg2* | ATGGGTGAAACTCTGGGAGATT | CGAAGTTGGTGGGCCAGAATGG |
| *Apob* | AAAGCTGCTCTCCAGGCCCTGA | TCTCCACGGGAGCGACACCATT |
| *Cd36* | TGACGTGGCAAAGAACAGCAGC | AGACACAGTGTGGTCCTCGGGG |
| **Bacteria** | | |
| *Bac* | AGAGTTTGATCCTGGCTCAG | GGTTACCTTGTTACGACTT |
| *BC* | GCATGGATACACTGATTGCCG | GGATACTCTGTTCCATCGCTGA |
| *aroA* | AGGCTTTCCTCCTTTACG | CCGCTCCAGTCACTTTCT |
| *aroB* | GACCGACGAGCATACCCA | TAGAAGCGGCGAAACCAC |
| *aroF* | CGTGTAGTGCGGAAAG | GCCACCTCAGTCATAACCCGAAGTC |
| *F1_BC* | GTTATCATCCGTGCTG | |
| *R1_pGERM* | TTCATAGGGAATAGAGTGT | |
| *F2_pGERM* | ACGCCAGGGTTTT | |
| *R2_BC* | GGCTCTTTCGTATCAT | |

**Fig. S1.** **Characteristics of the human cohort and gut microbial alterations in obesity and metabolic disorders.**

(A) Distribution of BMI across the human cohort (B) Differences in gut metagenomic α-diversity in the human cohort. (C) PLS-DA analysis based on the relative genus abundances of gut microbiota. (D) Relative abundance of the genus *Bacteroides* in healthy and overweight groups. (E) Relative abundance of *B. cellulosilyticus* in patients with metabolic diseases compared with healthy controls, and correlation analysis. Data presented as mean ± SEM. Compared with indicated group, **P* < 0.05, ***P* < 0.01, *****P* < 0.0001, ns: no significant.

**Fig. S2.** **Evaluation of *B. cellulosilyticus* colonization and its impact on metabolic phenotypes in HFD-fed mice.**

(A) Validation of *B. cellulosilyticus* colonization in the mouse gut by RT-qPCR analysis of fecal samples. (B) Food intake of mice. The blood glucose curves of the OGTT for each group of mice (C) and AUC value (D). (E) MASLD histological scoring criteria based on liver steatosis, inflammation, and ballooning. Data presented as mean ± SEM. Compared with indicated group, ***P* < 0.01, ****P* < 0.001, *****P* < 0.0001, ns: no significant.

**Fig. S3.** **Permutation test of the metabolomic OPLS-DA model and mediation analysis.**

Permutation test plots (n = 200) for the OPLS-DA models derived from fecal metabolomic data in negative ion mode **(A)** and positive ion mode **(B)**. (C) Mediation analysis of phenylalanine metabolites on ALT levels. (D) Mediation analysis of phenylalanine metabolites on AST levels. **P* < 0.05, ***P* < 0.01.

**Fig. S4. Dose-dependent effects of 2-HPAA on body weight and serum lipids in mice.**

(A) Body weight changes of mice during the experimental period. (B) Final body weight gain. Serum levels of TC (C)and TG (D) . Data are presented as mean ± SEM. Compared with indicated group, **P* < 0.05, ***P* < 0.01, ns: no significant.

**Fig. S5. 2HPAA regulates the gene expression profile and lipid composition of the liver.**

(A) Enrichment analysis of DEGs. (B) Expression of lipid transport/metabolism-related genes. (C-G) The relative expression levels of mRNA for key genes involved in lipid metabolism. (H) Lipidomic profiling of liver tissues. Data presented as mean ± SEM. Compared with indicated group, **P* < 0.05, ***P* < 0.01, ****P* < 0.001, *****P* < 0.0001.

**Fig.S6. Exogenous supplementation of 2HPAA can significantly alleviate the impaired glucose tolerance and hepatic steatosis induced by high-fat diet.**

(A-B) The blood glucose curves of the OGTT for each group of mice and AUC value. (C) MASLD histological scoring criteria based on liver steatosis, inflammation, and ballooning. Data presented as mean ± SEM. Compared with indicated group, ****P* < 0.001, *****P* < 0.0001.

**Fig. S7**. **Genomic characteristics and metabolic function validation of *B. cellulosilyticus*.**

(A) Whole-genome map of B. cellulosilyticus. (B) COG functional annotation for the genome of *B. cellulosilyticus*. (C) Schematic representation of the metabolic modules involved in 2HPAA biosynthesis. (D) Targeted metabolomic analysis of phenylalanine and tyrosine levels in feces. Data presented as mean ± SEM. Compared with indicated group, *****P* < 0.0001.

**Fig. S8. Construction and identification of the *B. c^∆aroB^* strain.**

(A) Schematic diagram illustrating the workflow for *aroB* gene deletion in *B.cellulosilyticus*. (B) Validation of pGERM::*aroB* insertion into *aroB* gene of *B.cellulosilyticus* genome. (C) Comparison of 2HPAA production levels among the blank control, *B. cellulosilyticus*, and *B.c*^Δ^*^aroB^*. Data are presented as mean ± SEM. Compared with indicated group, ***P* < 0.01.

**Fig. S9. Liver tissue single-cell sequencing annotation.**

(A) UMAP dimensionality reduction clustering plot of all cells. Different colors represent distinct cell subpopulations identified through unsupervised clustering. (B) Dot plot displaying expression patterns of representative marker genes for major cell types. Point size indicates gene expression proportion within cell populations, while color intensity reflects average expression levels. (C-D) Cell type scores for each cluster generated by the sc-type tool. (D) Feature plots illustrating the expression distribution of canonical marker genes for the 8 major cell types.

**Fig. S10. Marker feature plot of liver cell subtypes.**

(A) Feature plots illustrating the distribution of marker gene expression across 4 hepatocyte subtypes.

**Fig. S11. The results of the differential enrichment analysis of liver cell subtypes.**

(A-C) Enrichment analysis dot plot showing significantly enriched signaling pathways for differentially expressed genes across distinct cell subpopulations.

**Fig. S12. Molecular docking of 2HPAA with PPARγ.**

**Fig. S13. The effects of 2HPAA on the palmitic acid levels in the liver of mice and the lipid profiles of liver cells.**

(A) The content of palmitic acid in the liver. (B) PCA cluster analysis of the OA group and the 2HPAA group. (C) The VIP scores of the characteristic lipid molecules significantly enriched in the OA group and the 2HPAA group. (D) PCA cluster analysis of the 2HPAA group and the 2HPAA+Rosi group. (E) The VIP scores of the characteristic lipid molecules significantly enriched in the 2HPAA group and the 2HPAA+Rosi group. Compared with indicated group, ***P* < 0.01, *****P* < 0.0001.

**Fig. S14. Population cohort validation of the correlation between 2HPAA and MASLD.**

Correlation of BMI (A), ALT (B), AST (C), TG (D), Uric acid (E), and GGT (F) with the relative content of 2HPAA. **P* < 0.05.

**Fig. S15 The relative abundance of *B. cellulosilyticus* is significantly reduced following a 30-day voyage.**

(A) Schematic overview of the study cohort and experimental design. (B) Relative abundance of *B. cellulosilyticus* between the pre-voyage and post-voyage groups. Data presented as mean ± SEM. Compared with indicated group, *****P* < 0.0001.

**Fig. S16 Regulation of *B. cellulosilyticus* on gut microbiota composition and analysis of 2HPAA metabolic genes in HFD mice.**

(A) PCoA analysis based on metagenomic sequencing data. (B) The α-diversity of the gut microbiota. (C) Stacked plot of the relative abundance of microbiota at Phylum level in each group of mice. (D) The LEfSe analysis revealed the characteristic bacterial species that exhibited significant differences among the three groups. (E) The composition of microorganisms with the *hppD* gene, including the distribution at the phylum level and genus level. (F) The differences in abundance of key genes involved in the shikimate acid pathway. Data presented as mean ± SEM. Compared with indicated group, **P* < 0.05, ****P* < 0.001, ns: no significant.

**Fig. S17 *B. cellulosilyticus* intervention significantly increased the abundance of genes related to 2HPAA synthesis in the gut microbiota of mice.**

The relative abundance of the *aroA* (A), *aroB* (B) and *aroF* (C) gene in the gut microbiota. Data presented as mean ± SEM. Compared with indicated group, **P* < 0.05, ***P* < 0.01, ns: no significant.
